# Association between normalized lactate load and mortality in patients with septic shock: an analysis of the MIMIC-III database

**DOI:** 10.1186/s12871-021-01239-3

**Published:** 2021-01-12

**Authors:** Han Chen, Shu-Rong Gong, Rong-Guo Yu

**Affiliations:** Surgical Intensive Care Unit, Fujian Provincial Clinical College of Fujian Medical University, Fujian Provincial Hospital, Fuzhou, China

**Keywords:** Septic shock, Lactate, MIMIC-III, Mortality

## Abstract

**Background:**

An index of dynamic lactate change that incorporates both the magnitude of change and the time interval of such change, termed “normalized lactate load,” may reflect the hypoxic burden of septic shock. We aimed to evaluate the association between normalized lactate load and 28-day mortality in adult septic shock patients.

**Methods:**

Patients with septic shock were identified from the Medical Information Mart for Intensive Care (MIMIC)-III database. Lactate load was defined as the sum of the area under the curve (AUC) of serial lactate levels using the trapezoidal rule, and normalized lactate load was defined as the lactate load divided by time. Receiver-operating characteristic curves were constructed to determine the performance of initial lactate, maximum lactate and normalized lactate load in predicting 28-day mortality.

**Results:**

A total of 1371 septic shock patients were included, and the 28-day mortality was 39.8%. Non-survivors had significantly higher initial lactate (means ± standard deviations: 3.9 ± 2.9 vs. 2.8 ± 1.7 mmol/L), maximum lactate (5.8 ± 3.8 vs. 4.3 ± 2.2 mmol/L), lactate load (94.3 ± 71.8 vs. 61.1 ± 36.4 mmol·hr./L) and normalized lactate load (3.9 ± 3.0 vs. 2.5 ± 1.5 mmol/L, all *p* <  0.001). The AUCs of initial lactate, maximum lactate and normalized lactate load were 0.623 (95% confidence interval: 0.596–0.648, with a cut-off value of 4.4 mmol/L), 0.606 (0.580–0.632, with a cut-off value of 2.6 mmol/L) and 0.681 (0.656–0.706, with a cut-off value of 2.6 mmol/L), respectively. The AUC of normalized lactate load was significantly greater than both initial lactate and maximum lactate (all *p* <  0.001). In the multivariate logistic regression model, normalized lactate load was identified as an independent risk factor for 28-day mortality.

**Conclusions:**

Normalized lactate load is an independent risk factor for 28-day mortality in adult septic shock patients. Normalized lactate load had better accuracy than both initial and maximum lactate in determining the prognosis of septic shock patients.

## Background

Sepsis and septic shock remain the main causes of admission to the intensive care unit (ICU) and death in critically ill patients [1]. Serum lactate is considered an index of tissue hypoxia and/or a hypermetabolic state with enhanced glycolysis, which are commonly seen in sepsis and septic shock [2]. Besides, it has been shown by a large number of studies that an elevated lactate level is associated with increased mortality [3]. Therefore, a lactate-guided therapy with repeated lactate measurements has been recommended by the Surviving Sepsis Campaign (SSC) guidelines [4].

An elevated blood lactate concentration at any time point is a “static” index to reflect the balance in its production and clearance at that very moment. In contrast, a “dynamic” index can reflect the change of lactate homeostasis. In other words, it describes not only the magnitude but also the duration and trend over time. Some “dynamic” indices have been proposed to better predict outcomes. In the early 1980s, Vincent et al. introduced the concept of serial lactate measurement in circulatory shock patients. They found that survivors had at least a 10% decrease in lactate during the first 60 min of treatment [5]. The time variables in lactate kinetics were continued to be studied and further advocated for lactate-guided treatment protocols in the following years [6–12].

A new approach to examine the dynamic lactate changes has been proposed in the 2010s, which incorporates both the magnitude of change and the time interval of such change [13–16]. The dynamic change of lactate over time is plotted, and the area under the curve (AUC) represents the overall lactate burden, termed “lactate area” [14], “lactate area score” [15, 16] or “lactate load” [17]. With the AUC divided by the time interval, the result represents the averaged lactate load in this period, termed “time-weighted average lactate” [13] or “normalized lactate load” [17]. Such indices have been shown to be associated with worse outcomes in pediatric septic shock patients [14], post-cardiosurgical patients [17] and heterogeneous cohorts of critically ill patients [13]. In addition, adult septic shock patients were also investigated in two studies [15, 16]. However, these variables have not been validated in a large cohort of adult septic shock patients. In the present study, we aimed to evaluate the association between normalized lactate load and 28-day mortality in adult septic shock patients by analyzing data from a large critical care database.

## Methods

### Data source

Data were collected from the Medical Information Mart for Intensive Care-III (MIMIC-III) [18]. In brief, MIMIC-III database is maintained by the Laboratory for Computational Physiology at the Massachusetts Institute of Technology. It contains de-identified health-related data associated with over forty thousand patients who stayed in critical care units of the Beth Israel Deaconess Medical Center between 2001 and 2012. The establishment of the database was approved by the institutional review boards of the Massachusetts Institute of Technology (Cambridge, MA) and Beth Israel Deaconess Medical Center (Boston, MA). Consent was obtained for the original data collection and therefore waived for the present study by the Institutional Review Board of Fujian Provincial Hospital. Data were extracted by Dr. Han Chen and Dr. Shu-Rong Gong, who completed the online training course of the National Institutes of Health (certification number: HC 36014736, SRG 35606844). The study was designed and conducted in accordance with relevant guidelines and regulations (Declaration of Helsinki).

### Data extraction

**Fig. 1:**
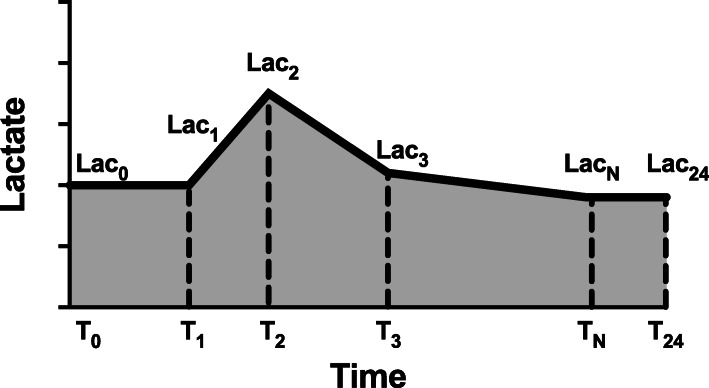
Diagram describing the calculation of lactate load and normalized lactate load. Each “T” on the x-axis represents the chart time of lactate measurement, while the corresponding “Lac” represents the corresponding lactate values. Lactate load was calculated as: ((Lac_1_ + Lac_0_)/2)) * (T_1_ - T_0_) + ((Lac_2_ + Lac_1_)/2)) * (T_2_ - T_1_) + … + ((Lac_24_ + Lac_N_)/2)) * (T_24_ - T_N_). Where the T_0_ represents ICU admission time, and the corresponding Lac_0_ was defined as equals to Lac_1_. Similarly, Lac_24_ (lactate value at 24 h after admission) was defined as equals to Lac_N_, where the Lac_N_ represents the last measured lactate value in the 24 h. Normalized lactate load was calculated as lactate load divided by (T_24_ – T_0_), which actually equals to 24 h

PostgreSQL tools Ver. 10 was used for data extraction. The following data were extracted by using Structured Query Language (SQL): age, gender, co-morbidities, length of ICU stay, sequential organ failure assessment (SOFA) score, vital signs, first-day lab results (such as white blood cell count, the levels of hemoglobin, platelet, bilirubin, blood urea nitrogen, creatinine and albumin), 28-day mortality, and the presence of septic shock. Besides, all arterial lactate values and the chart time of measurement were collected for further calculation. We used the term “lactate load” to represent the AUC of lactate, which accounts for the cumulative effect of hyperlactatemia over time. Meanwhile, we used the term “normalized lactate load” to represent the quotient of AUC divided by time, which accounts for the average intensity of hyperlactatemia. The calculation is detailed in Fig. 1.

Septic shock patients were screened for inclusion. The inclusion criteria were: 1) Patients fulfilled the definition of septic shock according to the sepsis-3.0 criteria [1]. 2) Patients have at least one lactate measurement within the first 24 h of ICU stay. The exclusion criteria were: 1) Length of ICU stay < 24 h. 2) Age less than 18 years. For patients who have multiple ICU admissions, only the first ICU stay was selected.

### Statistical analysis

Kolmogorov-Smirnov test was used for the assessment of the normality of distribution. Continuous variables were presented as mean with standard deviation (for normal distribution) or median with interquartile range (for non-normal distribution). Student’s *t*-test or Wilcoxon rank-sum test were used as appropriate. Categorical variables were presented as counts (percentages) and compared using the chi-square test. The correlation among maximum lactate, initial lactate and normalized lactate load was tested by the Pearson correlation analysis.

Univariate and multivariate logistic regression analyses with stepwise elimination were performed to evaluate the relationship between mortality and lactate variables. Variables with a *p*-value < 0.2 were included in the multivariate analysis. We artificially excluded creatinine and bilirubin because they are already included in the SOFA score. Receiver-operating characteristic (ROC) curves were constructed to determine the performance of initial lactate, maximum lactate, normalized lactate load and the SOFA score in predicting 28-day mortality. The Delong test was used to compare the AUCs of the ROC curves [19]. A subgroup analysis was performed in patients with a maximum lactate of ≥4 mmol/L since that lactate > 4 mmol/L has long been used as an indicator of tissue hypoperfusion [20]. STATA (ver. 15.1, StataCorp., TX, USA) and MedCalc (ver. 15.8, MedCalc Software, Ostend, Belgium) were used for data analysis. All reported *p-*values are two-sided, and a *p* <  0.05 was considered significant.

## Results

**Fig. 2:**
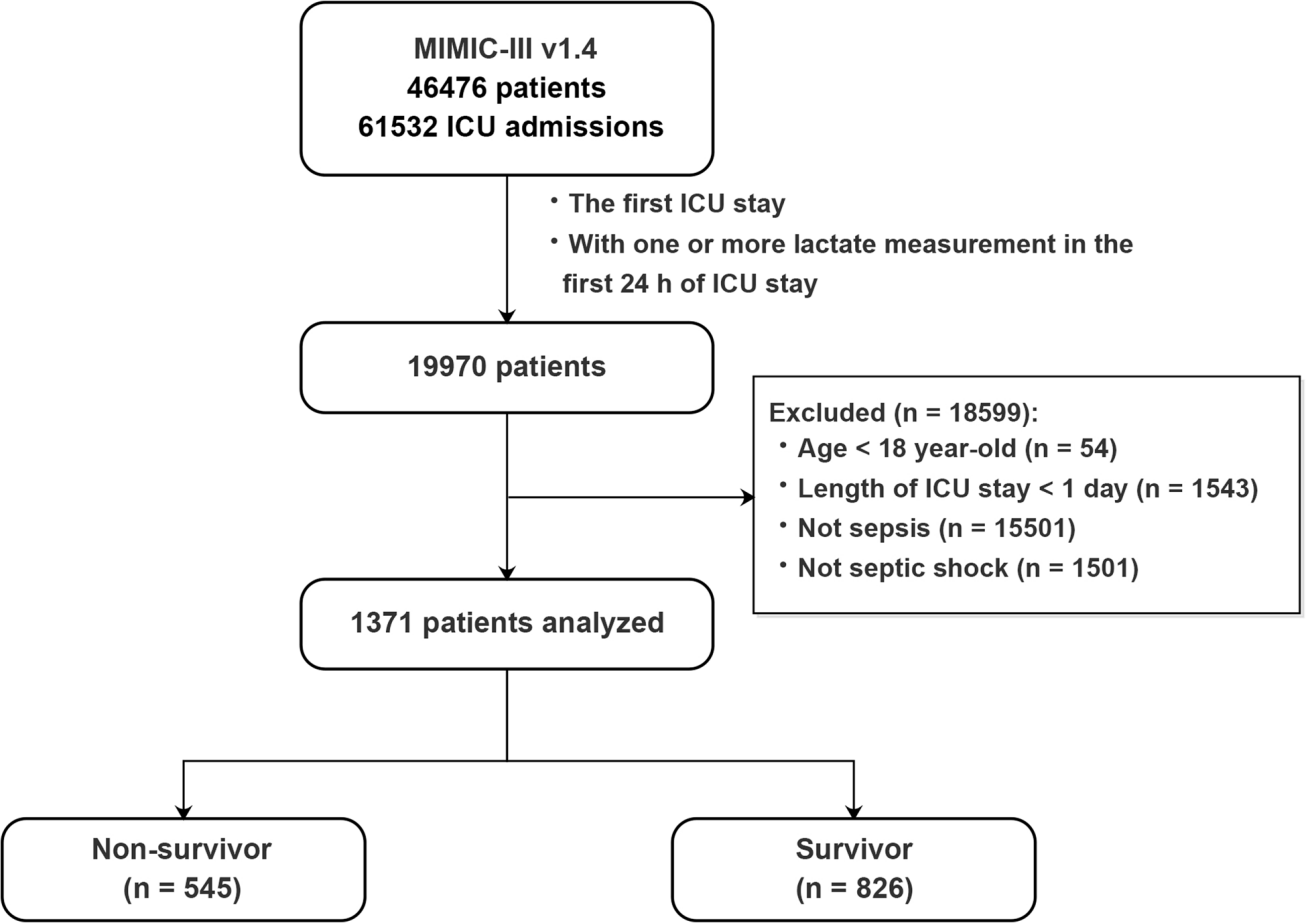
Flowchart showing a step-by-step selection of patients included in the study

A total of 1371 septic shock patients were included, and the 28-day mortality was 39.8% (826 survivors, 545 non-survivors, Fig. 2). Baseline patient characteristics are summarized in Table 1. In brief, non-survivors were older (66.5 ± 16.3 vs. 69.2 ± 14.7 years old, *p* = 0.001), and had higher SOFA score (8 [5, 10] vs. 10 [8, 13], *p* <  0.001). Maximum anion gap, maximum potassium, maximum bilirubin, maximum creatinine, maximum blood urea nitrogen, maximum activated partial thromboplastin time and maximum international normalized ratio were significantly higher in the non-survivors; whereas the maximum sodium, maximum chloride, minimum hematocrit, minimum albumin, minimum hemoglobin, minimum platelet were significantly lower in the non-survivors. In addition, non-survivors were more likely to have liver disease and malignant tumors.

**Table 1:** Comparisons of the clinical characteristics between survivors and non-survivors in the first 24 h

	Survivors (***n*** = 826)	Non-survivors (***n*** = 545)	***p*** value
Male	386 (46.7)	238 (43.7)	0.265
Age (years)	66.5 ± 16.3	69.2 ± 14.7	0.001
SOFA score	8 (5, 10)	10 (8, 13)	< 0.001
**Comorbidities**			
Obesity	51 (6.2)	28 (5.1)	0.420
Congestive heart failure	277 (33.5)	192 (35.2)	0.518
Cardiac arrhythmias	262 (31.7)	199 (36.5)	0.066
Valvular disease	91 (11)	61 (11.2)	0.919
Hypertension	139 (16.8)	101 (18.5)	0.416
Chronic pulmonary disease	162 (19.6)	109 (20)	0.860
Diabetes mellitus	282 (34.1)	182 (33.4)	0.775
Hypothyroidism	103 (12.5)	68 (12.5)	0.997
Liver disease	92 (11.1)	102 (18.7)	< 0.001
AIDS	4 (0.5)	0 (0)	0.104
Tumor	76 (9.2)	108 (19.8)	< 0.001
**Laboratory results in the first 24 h**			
Maximum anion gap (mmol/L)	18.9 ± 4.9	20.9 ± 6	< 0.001
Maximum sodium (mmol/L)	141.3 ± 6.4	140.6 ± 6.4	0.036
Maximum potassium (mmol/L)	4.8 ± 0.9	5.1 ± 1.1	< 0.001
Maximum chloride (mmol/L)	110.6 ± 7.4	108.9 ± 8.1	< 0.001
Maximum bilirubin (mg/L)	2.1 ± 4.2	3.9 ± 6.4	< 0.001
Maximum creatinine (mg/dL)	2.2 ± 1.9	2.5 ± 1.7	0.002
Maximum blood urea nitrogen (mg/dL)	39.4 ± 25.4	49.6 ± 30	< 0.001
Maximum blood glucose (mg/dL)	206.6 ± 119.9	207.8 ± 112.3	0.853
Minimum albumin (g/dL)	2.8 ± 0.7	2.5 ± 0.7	< 0.001
Minimum hematocrit (%)	29 ± 5.4	28.2 ± 5.9	0.008
Minimum hemoglobin (g/dL)	9.7 ± 1.8	9.3 ± 2	< 0.001
Minimum platelet (K/uL)	186.3 ± 122.6	159 ± 121.7	< 0.001
Maximum white blood cell count (K/uL)	19.8 ± 13.3	19.6 ± 23	0.835
Maximum APTT (sec)	47.1 ± 28.3	57.3 ± 34.8	< 0.001
Maximum INR (sec)	2 ± 1.5	2.5 ± 2.1	< 0.001
**Lactate related variables**			
Initial lactate (mmol/L)	2.8 ± 1.7	3.9 ± 2.9	< 0.001
Maximum lactate (mmol/L)	4.3 ± 2.2	5.8 ± 3.8	< 0.001
Lactate load (mmol·hr./L)	61.1 ± 36.4	94.3 ± 71.8	< 0.001
Normalized lactate load (mmol/L)	2.5 ± 1.5	3.9 ± 3.0	< 0.001

Data are presented as mean ± standard deviation or median (interquartile range) for continuous variables, and counts (percentages) for categorical variables

*AIDS* acquired immunodeficiency syndrome, *APTT* activated partial thromboplastin time, *INR* international normalized ratio, *SOFA* sequential organ failure assessment

Non-survivors had significantly higher initial lactate (3.9 ± 2.9 vs. 2.8 ± 1.7 mmol/L, *p* <  0.001), maximum lactate (5.8 ± 3.8 vs. 4.3 ± 2.2 mmol/L, *p* <  0.001), lactate load (94.3 ± 71.8 vs. 61.1 ± 36.4 mmol·hr./L, *p* <  0.001), and normalized lactate load (3.9 ± 3.0 vs. 2.5 ± 1.5 mmol/L, *p* <  0.001). There was a significant correlation between normalized lactate load and maximum lactate (*r* = 0.850, *p* <  0.001), and between normalized lactate load and initial lactate (*r* = 0.794, *p* <  0.001).

**Fig. 3:**
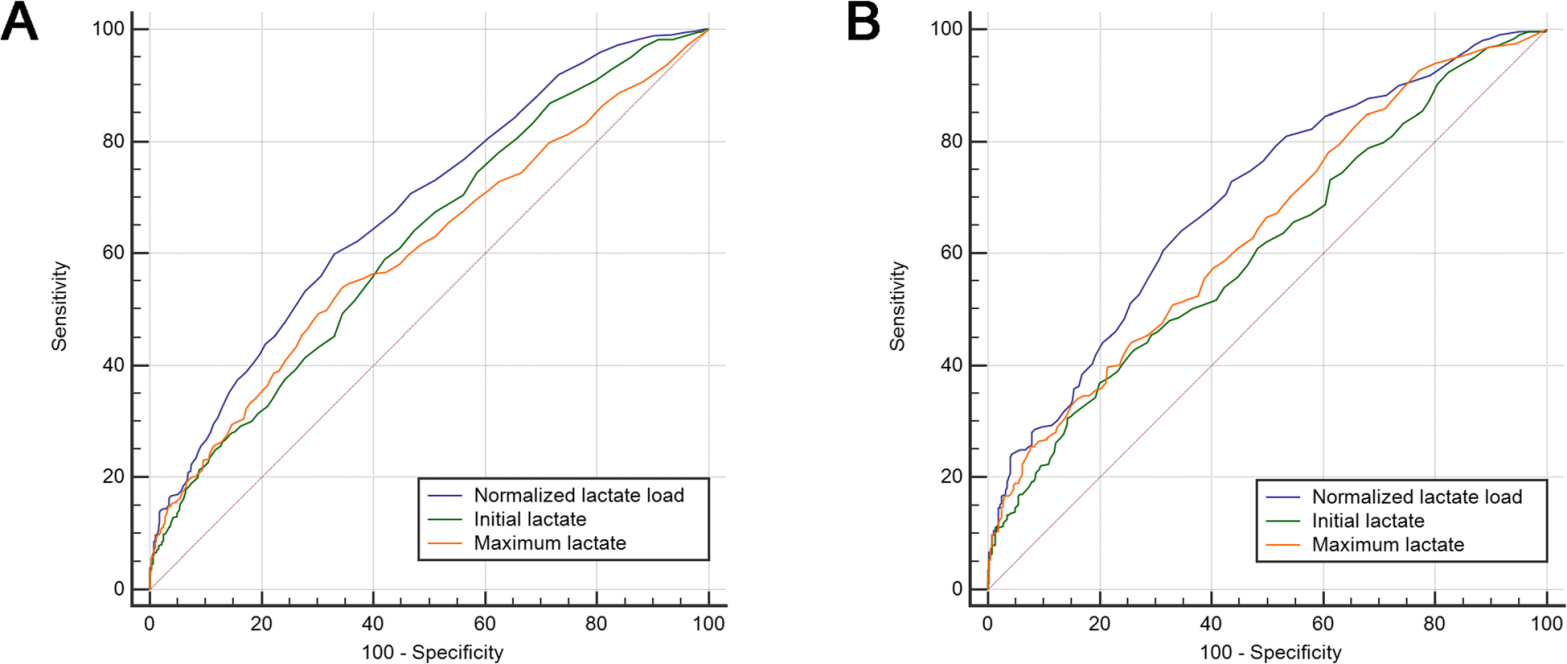
Comparisons among receiver-operating characteristic curves. Panel **a***:* Comparison of receiver-operating characteristic (ROC) curves between initial lactate, maximum lactate and normalized lactate load in all included patients. The AUC of normalized lactate load was significantly greater than the initial lactate and maximum lactate (all *p* <  0.001). There was no difference between initial lactate and maximum lactate (*p* = 0.207). Panel **b**: Comparison of ROC in patients with a maximum lactate ≥4 mmol/L. The AUC of normalized lactate load was significantly greater than both initial lactate and maximum lactate (*p* <  0.001 and *p* = 0.005, respectively). There was no difference between initial lactate and maximum lactate (*p* = 0.124)

We generated ROC curves for three lactate indicators (Fig. 3). Normalized lactate load had the strongest predictive power in both the overall population and the patients with greater maximum lactate (≥ 4 mmol/L, Table 2). The AUCs of initial lactate, maximum lactate and normalized lactate load were 0.623 (95% confidence interval [CI]: 0.596–0.648, with a cut-off value of 4.4 mmol/L), 0.606 (0.580–0.632, with a cut-off value of 2.6 mmol/L) and 0.681 (0.656–0.706, with a cut-off value of 2.6 mmol/L), respectively. The AUC of normalized lactate load was significantly greater than both initial lactate and maximum lactate (all *p* <  0.001). There was no difference between initial lactate and maximum lactate (*p* = 0.207). In the subgroup of patients with a maximum lactate of ≥4 mmol/L, the AUCs of initial lactate, maximum lactate and normalized lactate load were 0.609 (0.571–0.645, with a cut-off value of 5.1 mmol/L), 0.642 (0.604–0.678, with a cut-off value of 6.9 mmol/L) and 0.696 (0.660–0.730, with a cut-off value of 3.6 mmol/L). The AUC of normalized lactate load was significantly greater than both initial lactate and maximum lactate (*p* <  0.001 and *p* = 0.005, respectively). There was no difference between initial lactate and maximum lactate (*p* = 0.124). SOFA score had a similar predictive value to normalized lactate load in both the overall population (AUC 0.683 [0.658–0.708], *p* = 0.891) and patients with a maximum lactate of ≥4 mmol/L (AUC 0.706 [0.671–0.740], *p* = 0.617).

**Table 2:** Performance of normalized lactate load, initial lactate and maximum lactate in predicting mortality

	Cut-off value	Area under curve (95% CI)	Sensitivity (%, 95 CI)	Specificity (%, 95 CI)	Positive likelihood ratio (95% CI)	Negative likelihood ratio (95% CI)	Positive predictive value (95% CI)	Negative predictive value (95% CI)
**In overall population (*n*** **= 1371)**								
Normalized lactate load (mmol/L)	2.6	0.681 (0.656–0.706)	60 (55.8–64.1)	66.95 (63.6–70.2)	1.82 (1.6–2.0)	0.6 (0.5–0.7)	54.5 (50.4–58.5)	71.7 (68.4–74.9)
Initial lactate (mmol/L)	2.6	0.623 (0.596–0.648)	58.9 (54.6–63.1)	57.99 (54.5–61.4)	1.4 (1.3–1.6)	0.71 (0.6–0.8)	48.1 (44.2–51.9)	68.1 (64.6–71.6)
Maximum Lactate (mmol/L)	4.4	0.606 (0.580–0.632)	53.94 (49.7–58.2)	65.5 (62.1–68.7)	1.56 (1.4–1.8)	0.7 (0.6–0.8)	50.8 (46.6–54.9)	68.3 (64.9–71.5)
**In patients with a maximum lactate ≥ 4 mmol/L (*n*** **= 686)**								
Normalized lactate load (mmol/L)	3.6	0.696 (0.660–0.730)	64.04 (58.5–69.3)	65.31 (60.2–70.2)	1.85 (1.6–2.2)	0.55 (0.5–0.6)	61.3 (55.8–66.6)	67.9 (62.8–72.7)
Initial lactate (mmol/L)	5.1	0.609 (0.571–0.645)	36.91 (31.6–42.5)	79.95 (75.5–83.9)	1.84 (1.4–2.4)	0.79 (0.7–0.9)	61.3 (54.0–68.2)	59.6 (55.1–64.0)
Maximum Lactate (mmol/L)	6.9	0.642 (0.604–0.678)	44.16 (38.6–49.8)	74.25 (69.5–78.6)	1.72 (1.4–2.1)	0.75 (0.7–0.8)	59.6 (53.0–65.9)	60.8 (56.1–65.3)

*CI* confidence interval

In the multivariate logistic regression model, normalized lactate load, age, SOFA score, maximum anion gap, maximum chloride, maximum potassium, minimum hemoglobin, and the presence of malignant tumor were identified as independent risk factors of 28-day mortality (Table 3). For every 1 mmol/L increase in normalized lactate load the risk of 28-day mortality increased by 30% (odds ratio [OR] = 1.30, 95% CI 1.20 to 1.42, *p* <  0.001).

**Table 3:** Multivariate logistic regression models for the prediction of 28-day mortality

Effect	Odds ratio	95% CI	***p*** value
Normalized lactate load	1.30	(1.2, 1.42)	< 0.001
Age	1.03	(1.02, 1.03)	< 0.001
SOFA score	1.16	(1.12, 1.2)	< 0.001
Maximum anion gap	0.97	(0.94, 0.99)	0.036
Maximum chloride	0.97	(0.95, 0.98)	< 0.001
Maximum potassium	1.31	(1.15, 1.48)	< 0.001
Tumor	2.32	(1.63, 3.3)	< 0.001
Minimum hemoglobin	0.92	(0.86, 0.98)	0.030
Constant	0.44	(0.05, 3.62)	0.444

*SOFA* sequential organ failure assessment, *CI* confidence interval

## Discussion

In this study, we found that normalized lactate load was independently associated with 28-day mortality in a large cohort of septic shock patients. Normalized lactate load had better accuracy than both initial and maximum lactate in determining the prognosis of septic shock patients.

There is a large number of studies indicating that blood lactate concentration closely relates to survival of patients with septic shock [2, 21, 22]. However, a single isolated lactate level is not good enough for predicting the outcome or guiding therapy and the change of lactate could provide more information [5, 9, 23]. For this reason, the concept of dynamic change of lactate is attractive. Serial lactate and lactate clearance have been proposed to guide shock resuscitation [5, 9]. Lactate clearance can effectively reflect dynamic changes in lactate levels but cannot provide information regarding the severity of hyperlactatemia. The magnitude of organ dysfunction of septic shock depends upon the magnitude and duration of hypoxia. In this regard, the production of the actual lactate concentrations and the duration of hyperlactatemia can serve as a marker of organ hypoxia, which is the cause of organ dysfunction and death [2].

As previously mentioned, this index of lactate variation has various names. In this study, we adopted the terms “lactate load” and “normalized lactate load,” which were first proposed by Zhang et al. [17], to define lactate variation over time. By using the term “lactate load,” one can express the concept of hypoxic load or hypoxic burden in septic shock patients. Similarly, the term “normalized lactate load” reflects the “standardized” or the “averaged” hypoxic burden in the early phase of septic shock. Another advantage of using normalized lactate load is that it is easier to understand and apply in daily practice. Unlike the lactate area with a unit of “mmol·hr./L,” whose physiological meaning is difficult to understand, normalized lactate load is expressed in “mmol/L” (same as lactate concentration), and this makes it easy to interpret and allows one to compare it with a newly obtained lactate value directly.

Our data suggest that normalized lactate load may be an independent risk factor of mortality in septic shock patients. Although similar findings have been found in a few previous studies [13–16], it has not been investigated in a large cohort of adult septic shock patients like our study population. Nichol et al. found that time-weighted average lactate, which was defined as “summing the mean value between consecutive time points multiplied by the period of time between consecutive time points and then dividing by the total time”, was independently predictive of hospital mortality in a heterogeneous cohort of critically ill patients, with an OR of 1.37 (95% CI: 1.29 to 1.45) [13]. In the present study, we chose 28-day mortality as outcome, and our finding is close to theirs (OR = 1.30). Similarly, Yu et al. reported that early lactate area score, which was defined as “the sum of the AUC measured at 2, 4, 6, and 12 hours following the initial measurement”, was 0.659 in predicting 28-day mortality, which is also close to our findings. On the other hand, Kim et al. reported a higher AUC (AUC = 0.828) of the lactate area (defined as the sum of the AUC of serial lactate levels measured during the 24 h following admission) for 28-day mortality. In their study, 65 pediatric patients with septic shock were included, and the overall 28-day mortality was 26.2% [14]. In addition, Wang et al. also reported a higher AUC of lactate area score (defined as the sum of the AUC of serial lactate levels measured during the 24 h following admission divided by 24) in predicting 28-day mortality (AUC = 0.758). In their study, 115 elderly (age ≥ 65 years) patients with septic shock were included, and the overall 28-day mortality rate was 67.0%. Apart from the difference in study populations and sample sizes, one more important difference between our study and previous studies is that we reported not only lactate load (like the previous studies, although the terms may differ) but also normalized lactate load, which was not always included in previous studies.

Several limitations in the present study should be considered. First, our study was a retrospective study based on electronic healthcare records, and therefore limited by the nature of the retrospective design and the source of data used. Second, there was not a standard protocol of lactate measurement in this study, and it is possible that lactate load and normalized lactate load were underestimated or overestimated. The lack of a standard lactate measurement protocol also precluded the calculation of lactate clearance, which is another traditionally employed index of lactate variation [6, 11]. However, our results simply reflect the actual effect of normalized lactate load measurement in real-world clinical practice. Third, for patients with a single lactate measurement, normalized lactate load was equal to their single lactate measurement and this may also underestimate or overestimate lactate load and normalized lactate load. Among the 241 (17.6%) patients who had only a single lactate measurement, 215 (89.2% of the 241 patients and 15.7% of the whole study population) had a lactate value of ≤4 mmol/L. Clinicians may have considered a lactate ≤4 mmol/L to be acceptable in the absence of other evidence of tissue hypoxia. Notably, lactate > 4 mmol/L significantly increases ICU admission rates and mortality rates [9, 24]. Fourth, normalized lactate area cannot distinguish a decreasing or increasing pattern of lactate kinetic change. Finally, The AUC, sensitivity and specificity, along with the negative and positive predictive value of normalized lactate load were not very high. Interestingly, we found similar AUCs between normalized lactate load and SOFA score (both are not satisfactory enough). A single indicator cannot accurately predict the prognosis in a highly heterogeneous population like septic shock patients and should be integrated with a variety of clinical manifestations, laboratory exams and imaging.

## Conclusions

Normalized lactate load is independently associated with 28-day mortality in adult septic shock patients. Normalized lactate load had better accuracy than both initial and maximum lactate in determining the prognosis of septic shock patients.

## Data Availability

The data that support the findings of this study are available from the MIMIC-III database, but restrictions apply to the availability of these data, which were used under license for the current study, and so are not publicly available. Data are however available from the authors upon reasonable request and with permission of the holder of the database.

## Abbreviations

AUC: Area under curve
CI: Confidence interval
ICU: Intensive care unit
MIMIC: Medical Information Mart for Intensive Care
OR: Odds ratio
ROC: Receiver-operating characteristic
SOFA: Sequential organ failure assessment
SQL: Structured Query Language
SSC: Surviving Sepsis Campaign
